# Inositol 1,4,5-trisphosphate receptors are essential for fetal-maternal connection and embryo viability

**DOI:** 10.1371/journal.pgen.1008739

**Published:** 2020-04-22

**Authors:** Feili Yang, Lei Huang, Alexandria Tso, Hong Wang, Li Cui, Lizhu Lin, Xiaohong Wang, Mingming Ren, Xi Fang, Jie Liu, Zhen Han, Ju Chen, Kunfu Ouyang

**Affiliations:** 1 School of Chemical Biology and Biotechnology, State Key Laboratory of Chemical Oncogenomics, Peking University Shenzhen Graduate School, Shenzhen, China; 2 Department of Cardiovascular Surgery, Peking University Shenzhen Hospital, Shenzhen, China; 3 University of California San Diego, School of Medicine, Department of Medicine, La Jolla, CA, United States of America; 4 Department of Pharmacology, Tianjin Key Laboratory of Inflammation Biology, School of Basic Medical Sciences, Tianjin Medical University, Tianjin, China; 5 Department of Pathophysiology, School of Medicine, Shenzhen University, Shenzhen, China; Indiana University Purdue University at Indianapolis, UNITED STATES

## Abstract

Inositol 1,4,5‐trisphosphate receptors (IP_3_Rs) are a family of intracellular Ca^2+^ release channels located on the ER membrane, which in mammals consist of 3 different subtypes (IP_3_R1, IP_3_R2, and IP_3_R3) encoded by 3 genes, *Itpr1*, *Itpr2*, and *Itpr3*, respectively. Studies utilizing genetic knockout mouse models have demonstrated that IP_3_Rs are essential for embryonic survival in a redundant manner. Deletion of both IP_3_R1 and IP_3_R2 has been shown to cause cardiovascular defects and embryonic lethality. However, it remains unknown which cell types account for the cardiovascular defects in IP_3_R1 and IP_3_R2 double knockout (DKO) mice. In this study, we generated conditional IP_3_R1 and IP_3_R2 knockout mouse models with both genes deleted in specific cardiovascular cell lineages. Our results revealed that deletion of IP_3_R1 and IP_3_R2 in cardiomyocytes by TnT-Cre, in endothelial / hematopoietic cells by Tie2-Cre and Flk1-Cre, or in early precursors of the cardiovascular lineages by Mesp1-Cre, resulted in no phenotypes. This demonstrated that deletion of both IP_3_R genes in cardiovascular cell lineages cannot account for the cardiovascular defects and embryonic lethality observed in DKO mice. We then revisited and performed more detailed phenotypic analysis in DKO embryos, and found that DKO embryos developed cardiovascular defects including reduced size of aortas, enlarged cardiac chambers, as well as growth retardation at embryonic day (E) 9.5, but in varied degrees of severity. Interestingly, we also observed allantoic-placental defects including reduced sizes of umbilical vessels and reduced depth of placental labyrinth in DKO embryos, which could occur independently from other phenotypes in DKO embryos even without obvious growth retardation. Furthermore, deletion of both IP_3_R1 and IP_3_R2 by the epiblast-specific Meox2-Cre, which targets all the fetal tissues and extraembryonic mesoderm but not extraembryonic trophoblast cells, also resulted in embryonic lethality and similar allantoic-placental defects. Taken together, our results demonstrated that IP_3_R1 and IP_3_R2 play an essential and redundant role in maintaining the integrity of fetal-maternal connection and embryonic viability.

## Author summary

Inositol 1,4,5‐trisphosphate receptors (IP_3_Rs) are a family of intracellular Ca^2+^ release channels located on the ER membrane, which in mammals consist of 3 different subtypes (IP_3_R1, IP_3_R2, and IP_3_R3). IP_3_R-mediated Ca^2+^ signaling has been proposed to play an essential role in regulating cardiovascular development, and IP_3_R1 and IP_3_R2 double knockout (DKO) mice has been shown to develop cardiovascular defects and embryonic lethality. However, our present study using conditional cell-specific gene deletion strategy revealed that deletion of both genes in cardiomyocytes, endothelial / hematopoietic cells, and early precursors of the cardiovascular lineages in mice could not result in similar lethal phenotypes. By contrast, we observed allantoic-placental defects including reduced sizes of umbilical vessels and reduced depth of placental labyrinth in DKO embryos, which could occur independently from other phenotypes in DKO embryos. We further found that deletion of both IP_3_R1 and IP_3_R2 in epiblast also resulted in embryonic lethality and similar allantoic-placental defects. Our results demonstrated that IP_3_R1 and IP_3_R2 play an essential and redundant role in maintaining the integrity of fetal-maternal connection and embryonic viability.

## Introduction

Inositol 1,4,5-trisphosphate (IP_3_) receptors (IP_3_Rs) are a family of intracellular Ca^2+^ release channels located on the membrane of endoplasmic reticulum (ER), which mediate Ca^2+^ mobilization from the ER to the cytoplasm when the receptors bind to the secondary messenger IP_3_ [1]. Three different subtypes of IP_3_Rs have been identified in mammals (IP_3_R1, IP_3_R2, and IP_3_R3) [2]. Using gene-knockout mouse models, IP_3_Rs have been shown to play an essential role in regulating diverse physiological processes, including brain function [3], taste perception [4], embryonic survival [5–7], extra-embryonic vascular development [7], exocrine secretion [8], T cell development [9], B cell function [10], gastrointestinal motility [11], vascular contractility and hypertension [12,13]. Interestingly, many of these studies demonstrated that IP_3_Rs are ubiquitously expressed and may function in a mechanism of redundancy between different subtypes [5–10,12,13]. In particular, IP_3_R1 and IP_3_R2 have been implied to play a role in regulating embryonic cardiac development. Deletion of both IP_3_R1 and IP_3_R2 caused developmental defects of ventricular myocardium and atrioventricular canal of the hearts, and embryonic lethality [6]. Since IP_3_R expression has been shown to occur before the appearance of ryanodine receptors in the early embryo [14], IP_3_R has been long proposed to drive the first cycling of Ca^2+^ within the heart and thus regulate embryonic cardiac development [15]. However, whether IP_3_R-mediated Ca^2+^ signaling was required for normal cardiac development and whether loss of IP_3_Rs in cardiovascular cell lineages could account for embryonic lethality of the IP_3_R1 and IP_3_R2 double knockout (DKO) mice remain unknown.

To address this question, we generated both conventional and tissue-specific IP_3_R1 and IP_3_R2 double knockout mouse models. Our results revealed that deletion of IP_3_R1 and IP_3_R2 in cardiomyocytes by TnT-Cre, in endothelial / hematopoietic cells by Tie2-Cre and Flk1-Cre, or in early precursors of the cardiovascular lineages by Mesp1-Cre, resulted in no embryonic lethal phenotypes, demonstrating that IP_3_R1 and IP_3_R2 in cardiovascular cell lineages are dispensable for embryonic survival. Subsequently, we observed the allantoic-placental defects that could occur independently from other phenotypes in DKO embryos. The placenta is a vital organ, which sits at the interface between the maternal and fetal circulation and facilitates the transport of nutrients and oxygen into the fetus [16]. The placenta is essential for survival and growth of the fetus during gestation, and placental dysfunction has been shown to result in various pregnancy diseases, fetal growth restriction, other pregnancy-associated disorders, and even embryonic death [17,18]. We found that the DKO embryos displayed reduced sizes of umbilical vessels and reduced depth of placental labyrinth. Furthermore, we generated a conditional IP_3_R1 and IP_3_R2 knockout mouse line utilizing the epiblast-specific Meox2-Cre (cKO^Meox2^), which targets all the fetal tissues and extraembryonic mesoderm but not extraembryonic trophoblast cells. The cKO^Meox2^ embryos displayed embryonic lethality and allantoic-placental defects, similar as DKO embryos, suggesting that epiblast cell lineages could, at least partially, account for the phenotypes observed in global IP_3_R1 and IP_3_R2 double knockout embryos. Taken together, our results demonstrated that IP_3_R1 and IP_3_R2 play an essential role in maintaining the integrity of fetal-maternal connection and embryonic viability.

## Materials and methods

### Mice

The generation of *Itpr1* mutant (*Itpr1*^-/-^), *Itpr1* floxed (*Itpr1*^f/f^), and *Itpr2* mutant (*Itpr2*^-/-^) mice has been previously described, respectively [9,19,20]. The *Itpr1*^+/-^*Itpr2*^-/-^ mice were generated and further intercrossed with each other to generate the global IP_3_R1 and IP_3_R2 double knockout (*Itpr1*^-/-^*Itpr2*^-/-^, DKO) and control (*Itpr1*^+/+^*Itpr2*^-/-^) mice. On the other hand, the *Itpr1*^f/f^*Itpr2*^-/-^ mice were crossed with the Tg(Tnnt2-cre)5Blh/JiaoJ (TnT-Cre; Jackson Laboratory) mice that express the Cre recombinase under the control of the rat cardiac troponin T2 [21], the B6.Cg-Tg(Tek-cre)1Ywa/J (Tie2-Cre; Jackson Laboratory) mice that have the mouse endothelial-specific receptor tyrosine kinase promoter directing expression of Cre recombinase [22], the Kdrtm1(cre)Sato/J (Flk1-Cre; Jackson Laboratory) mice that express the Cre recombinase under the control of the kinase insert domain protein receptor gene [23], the ICR.Cg-Mesp1tm2(cre)Ysa/YsaRbrc (Mesp1-Cre; International Mouse Strain Resource) mice that express the Cre recombinase under the control of endogenous promoter-enhancer [24], and the B6.129S4-Meox2tm1(cre)Sor/J (Meox2-Cre; Jackson Laboratory) mice that express the Cre recombinase under the control of the endogenous *Meox2* promoter [25], to generate the cell / tissue-specific IP_3_R1 and IP_3_R2 double knockout mice, respectively. Briefly, male Cre^+^*Itpr1*^f/+^*Itpr2*^-/-^ mice were generated and then crossed with female Cre^-^*Itpr1*^f/f^*Itpr2*^-/-^ mice for examination of the vaginal plug. Genotypic analysis was first performed at postnatal day 1 to see whether the offspring from each cross were born at Mendelian ratios. If embryonic lethality was suspected, embryos were then dissected at various embryonic stages. Otherwise, embryos were only collected at embryonic day (E) 10.5 for morphological analysis. Mesp1-Cre and Meox2-Cre mice were also crossed with the B6.129S4-Gt(ROSA)26Sortm1Sor/J (Rosa-LacZ; Jackson Laboratory) mice to generate Mesp1-Cre/Rosa-LacZ and Meox2-Cre/Rosa-LacZ mice for cell lineage tracing analysis, respectively.

### DNA analysis

Genomic DNA was extracted from mouse tails or embryonic yolk sacs as previously described [26], and polymerase chain reaction (PCR) was utilized to genotype the mice using the following gene-specific primers (from 5’ to 3’): *Itpr1* wildtype and floxed allele (forward, AGACCTCTGCCTTAGGA GGTATTT; reverse, ACTGGGCAGGCATATATAGTTAGC), *Itpr1* mutant allele (forward, AGACCTCTG CCTTAGGAGGTATTT; reverse, TTTAAGAAAGCAAGGAGAAGGAGA), *Itpr2* wildtype allele (forward, GCTGTGCCCAAAATCCTAGCACTG; reverse, CATGCAGAGGTCGTGTCAGTCATT), mutant allele (forward, AATGGGCTGACCGCTTCCTCGT; reverse, AGTGATACAGGGCAAGTTCATAC), *TnT-Cre* / *Flk1-Cre* (forward, GAGCATACCTGGAAAATGCTTC; reverse, CCGGCAAAACAGGTAGTTATTC), *Tie2-Cre* (forward, CCCTGTGCTCAGACAGAAATGAGA; reverse, CGCATAACCAGTGA AACAGCATTGC), *Mesp1-Cre* (forward, CTCTGAGCATGGTTCTTTCAAC; reverse, TCCCTGAA CATGTCCATCAGGTTC), Meox2-Cre (forward, ACCTCTCCCACACTTGACATCT; reverse, GAAGCATTTTCCAGGTATGCTC), *Rosa-Laz* (forward 1, AAAGTCGCTCTGAGTTGTTAT; forward 2, GCGAAGAGTTTGTCCTCAACC; reverse, GGAGCGGGAGAAATGGATATG).

### Quantitative real time PCR analysis

The hearts and blood cells were collected from mouse embryos at E10.5, and total RNA was extracted using TRIzol reagent (Invitrogen). cDNA was synthesized using the TransScript One-Step cDNA Synthesis SuperMix Kit (Transgen Biotech). Quantitative real time PCR was then performed using TransStart Tip Green qPCR SuperMix (Transgen Biotech) according to the manufacturer’s instruction. The sequences for primers of *Itpr1* and *Gapdh* were used as previously described [27]. Relative transcript abundance was normalized to *Gapdh*. Each sample was run at least in duplicate.

### Morphological and histological analysis

Mice were mated under the standard 12-hour light / dark cycle, and noon on the day of the appearance of the vaginal plug was defined as the embryonic day 0.5 (E0.5). Embryos and placentas were dissected and collected under a Leica MZ6 dissecting light microscope and photographed as previously described [28]. The tissues were then fixed in 4% paraformaldehyde (PFA) diluted in phosphate buffered saline (PBS). For paraffin sections, the tissues were dehydrated through ethanol gradients and xylene, and embedded in paraffin. Serial sections (7 μm thick) were obtained and stained with hematoxylin and eosin (H&E) as previously described [29]. For frozen sections, tissues were incubated in a graded series of sucrose concentrations from 15% to 25%, and then embedded in optimal cutting temperature (OCT) compound (Sakura Finetek USA Inc., Torrance, CA, USA).

### Whole-mount RNA in situ hybridization

In situ hybridization was performed using digoxigenin-labeled antisense riboprobes against *Itpr1* (nucleotides 5051–6199, Genbank accession no. NM_010585.2), *Itpr2* (nucleotides 4629–5644, Genbank accession no. NM_010586.1), and *Itpr3* (nucleotides 7974–8733, Genbank, NM_080553.2). Briefly, embryos were collected and fixed in 4% PFA overnight, and then washed twice with PBS containing 0.1% Tween-20 (PBT). The embryos were dehydrated through a series of PBT-methanol washes and rehydrated through a reciprocal series of PBT-methanol washes. The embryos were then treated with 6% hydrogen peroxide diluted in PBT for 1 hour, followed by the digestion with 10μg/ml proteinase at room temperature for varied times depending on the stage of embryos. The digestion was stopped by 2 mg/ml glycine diluted in PBT. The samples were then post-fixed in 4% PFA, 0.2% glutaraldehyde and 0.1% Tween-20 for 20 minutes at room temperature, and prehybridized 2 hours in hybridization solution (50% formamide, 5X SSC, 1% SDS, 100 μg/ml yeast tRNA, 50 μg/ml heparin) at 65°C. After that, the embryos were incubated with fresh hybridization solution containing the digoxigenin-conjugated riboprobe at 65°C with rocking overnight. After hybridization, the embryos were washed twice with solution I (50% formamide, 5X SSC, 1% SDS) at 65°C (for 30 minutes each time), twice with solution II (50% formamide, 2X SSC) at 65°C (for 30 minutes each time), and then with TBST containing 140 mM NaCl, 2.5 mM KCl, 25 mM Tris, and 0.1% Tween-20. The embryos were then incubated with a blocking solution containing 1% blocking reagent at room temperature, followed by another incubation with a fresh blocking solution containing the anti-digoxigenin AP-conjugated antibody at 4°C. Finally, signals were detected using the NBT/BCIP solution and photographed under a stereomicroscope.

### RNA in situ hybridization in cryosections

RNA in situ hybridization in cryosections was performed as previously described [30]. The probes against *Hand1*, *Csh1* and *Dlx3* are a kind gift from J.C. Cross. Sections (10 μm) were briefly rehydrated in PBS, post-fixed in 4% PFA, and treated with proteinase K (15 μg/ml) for various times depending on the ages of the embryos. The samples were then acetylated for 10 minutes in 0.25% (v/v) acetic anhydride, and hybridized with digoxigenin-conjugated riboprobes overnight at 65°C in sealed humidified boxes. After hybridization, the sections were treated with RNase, incubated with the blocking solution, and then treated with anti-digoxigenin AP-conjugated antibody. Signals were detected using the NBT/BCIP solution and photographed.

### Whole-mount PECAM staining

Whole-mount PECAM staining was performed as previously described [28]. Briefly, the embryos dissected in ice cold PBS were fixed in 4% PFA overnight at 4°C. After fixation, the embryos were dehydrated in a graded methanol series (methanol concentrations of 25, 50, 75, and 100%, for 15 minutes each) at room temperature. After bleaching with 5% hydrogen peroxide in methanol for 4–5 hours, the embryos were then rehydrated using 75, 50, and 25% methanol and washed with PBS. After that, the embryos were incubated with the blocking solution for several hours at room temperature, and subsequently with the primary CD31 antibody (BD Pharmingen, catalog no. 550274) at 4°C overnight, followed by the incubation with the HRP-conjugated goat anti-rat IgG (Zsbio, catalog no.ZB2307) at 4°C overnight. The signals were developed using 3’,3’-diaminobenzidine, and photographed under a stereomicroscope.

### X-gal staining

For whole-mount X-gal staining, mouse embryos and placentas were freshly collected and fixed in 4% PFA at 4°C for 1 hour, and then washed three times, each time with cold PBS for 10 minutes. The samples were subsequently stained in the X-gal solution containing 5 mM ferrocyanide, 5 mM ferricyanide, 2 mM MgCl_2_, 1 mg/ml X-gal, 0.01% sodium deoxycholate, 0.02% NP-40, for several hours at 37°C.

For cryosections, the tissues were freshly collected, fixed in 4% PFA, and embedded in OCT compound. Cryosections (10 μm) were prepared and stained with the X-gal solution. Nuclear Fast Red was used to counterstain the sections.

### Transmission electron microscopy

Placentas were fixed with 2% PFA and 2% glutaraldehyde in 0.1 M phosphate buffer (pH 7.3). Specimens were post-fixed with 2% osmium tetroxide for 2 hours, stained with 3% uranyl acetate for 2 hours, and embedded in epoxy resin. Thin sections were cut, stained with uranyl acetate and lead citrate, and examined with transmission electron microscope.

### Statistics

Scatter diagrams were drawn using Graphpad Prism 5. Statistical analysis was performed using a two-tailed, unpaired Student’s *t* test as previously described [31]. All data represent mean ± SEM (error bars). *P* < 0.05 was considered statistically significant.

### Ethics statement

All mice were housed under a 12-hour day/night cycle at a temperature of 25°C. All animal care and experiments were conducted in accordance with the guidelines established by the Animal Care and Use Committee (IACUC) at Peking University Shenzhen Graduate School (Shenzhen, China), and approved by the IACUC (Approval #: AP0017). Periodic review of procedures was performed, and amendments were made as needed.

## Results

### Deletion of both IP_3_R1 and IP_3_R2 in mice caused growth retardation and embryonic lethality

To understand the role of IP_3_Rs in embryonic development, we first assessed expression of IP_3_R subtypes during early stages of embryonic development using RNA in situ hybridization (**S1 Fig**). IP_3_R subtypes were expressed in an overlapping fashion in mouse embryos including endocardium and atrioventricular cushions. In addition, distinct IP_3_R expression patterns were also observed in other embryonic domains, including pharyngeal arch arteries, sinus venosus, aortic endothelium, and forelimb (**S1 Fig**).

**Fig 1:**
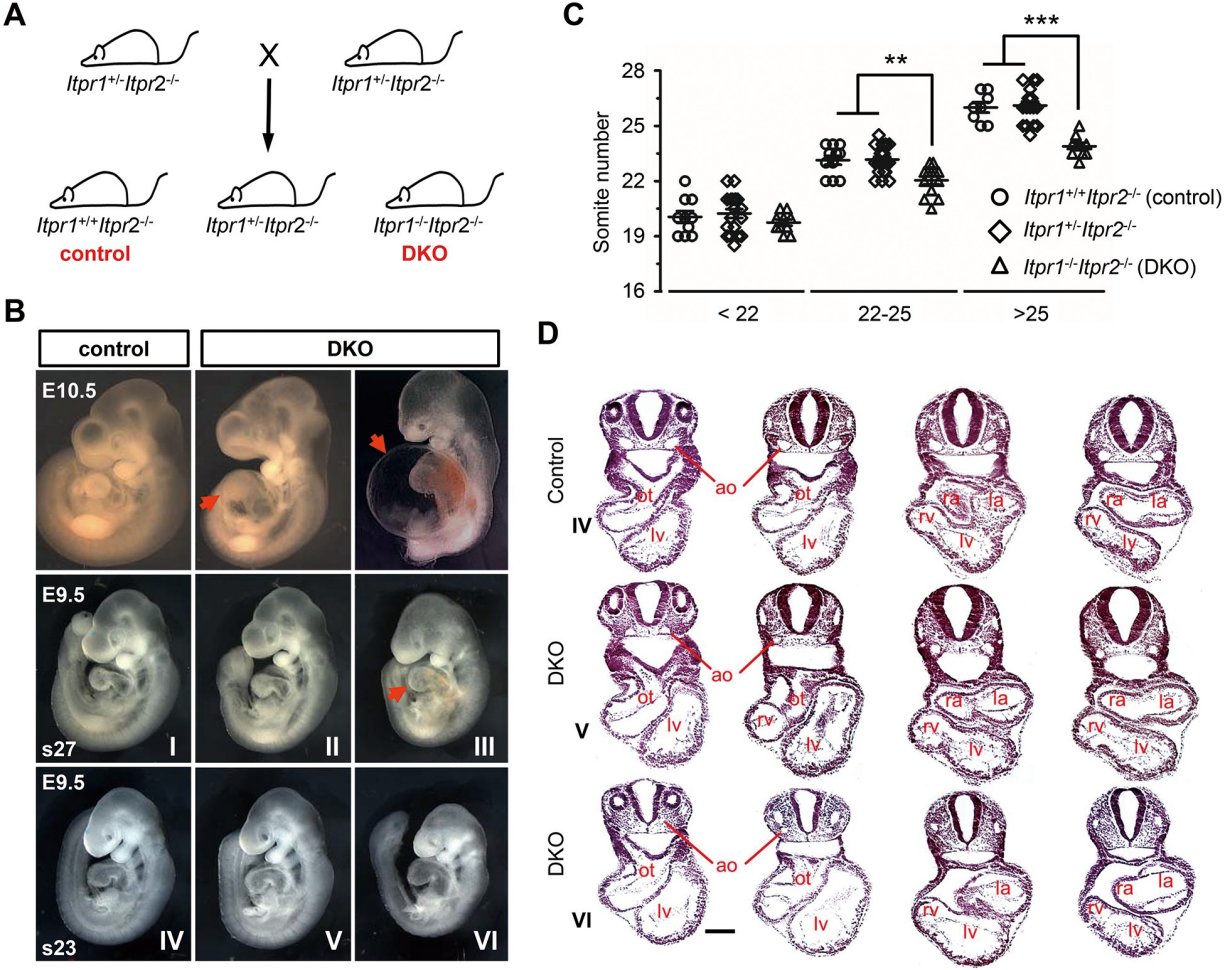
Generation and characterization of IP_3_R1 and IP_3_R2 double knockout mice. (**A**) Schematic diagram of mouse breeding strategy used to generate *Itpr1*^-/-^*Itpr2*^-/-^ (DKO) mice. The littermate *Itpr1*^+/+^*Itpr2*^-/-^ mice were used as control. (**B**) Whole mount assessment of DKO and control mouse embryos at the stages of E10.5 and E9.5. At E9.5, somite pair-matched DKO and control embryos were collected at the stages of 27 somites (s27) and 23 somites (s23), respectively. Please note the varying degrees of growth retardation in DKO embryos at the stages of s27 (II, III) and s23 (V, VI) when compared to littermate controls (I, IV). Red arrows denote chest edema observed in both DKO embryos at E10.5 and one DKO embryo at the stage of s27. (**C**) Statistic analysis of somite pairs in DKO embryos and the littermate control and *Itpr1*^+/-^*Itpr2*^-/-^ embryos at E9.5. According to the somite pair of control embryos, all embryos were divided into three groups, less than 22 (<22), between 22 and 25 (22–25), and above 25 (>25). n = 8–21 embryos for each group. All data represent mean ± SEM. Significance was determined by performing a two-tailed, unpaired Student’s *t*-test. **p < 0.01, ***p < 0.001 versus control. (**D**) Histological assessment of sectioned mouse embryos at the stage of s23 as shown in (B) following hematoxylin and eosin (H&E) staining. Chamber enlargement and wall thinning were also observed in DKO embryos (VI). ot, outflow tract; ao, aorta; ra, right atria; la, left atria; rv, right ventricle; lv, left ventricle. Black bar represents 0.25 mm.

We then generated global IP_3_R1 and IP_3_R2 double knockout (*Itpr1*^*-/-*^*Itpr2*^*-/-*^, DKO) mouse model by intercrossing phenotypically normal *Itpr1*^*+/-*^*Itpr2*^*-/-*^ mice (**Fig 1A**). Genotypic analysis revealed that no live DKO pups or embryos could be found after the embryonic day 14.5 (E14.5; **Table 1**). At E11.5, 4 of 44 embryos were identified as DKO pups, but all 4 of these DKO embryos were dead, developing no heart beat and undergoing reabsorption. On the other hand, DKO embryos were observed at the expected Mendelian ratio at E10.5, indicating that DKO embryos die sometime between E10.5 and E11.5, which is consistent with a previous study [6]. However, all DKO embryos at E10.5 developed apparent growth retardation and enlarged ventricles (**Fig 1B**), and most (21 off 23) DKO embryos developed chest edema, indicating cardiovascular dysfunction (**Fig 1B**). At E9.5, DKO embryos also exhibited growth retardation but with varied degrees when compared with their somite pair-matched control embryos (**Fig 1B**). The number of somite pairs in DKO embryos was significantly lower when their littermate control or *Itpr1*^+/-^*Itpr2*^-/-^ embryos had somite pairs between 22–24 or above 25. On the other hand, numbers of somite pairs in DKO embryos were comparable when their littermate control embryos had less than 22 somite pairs (**Fig 1C**), thus indicating an age-dependent growth retardation in DKO embryos. In addition, histological assessment in control and DKO embryos at the stage of 23–24 somite pairs also revealed that DKO embryos with obvious growth retardation (3 off 11 DKO embryos) exhibited both enlarged cardiac chambers and reduced size of aortas when compared with control embryos, whereas DKO embryos with no obvious growth retardation at the same stage only exhibited reduced size of aortas (**Fig 1D**).

**Table 1:** Genotypic analysis of embryos from *Itpr1*^+/-^*Itpr2*^-/-^ x *Itpr1*^+/-^*Itpr2*^-/-^ intercrosses.

Day of analysis	No. of genotype			Total
	*Itpr1*^+/+^*Itpr2*^-/-^	*Itpr1*^+/-^*Itpr2*^-/-^	*Itpr1*^-/-^*Itpr2*^-/-^	
E8.5	15 (24.6%)	32 (52.5%)	14 (23.0%)	61
E9.5	55 (24.9%)	109 (49.3%)	57 (25.8%) ^a^	221
E10.5	27 (24.5%)	60 (54.5%)	23 (20.9%) ^b^	110
E11.5	12 (27.3%)	28 (63.6%)	4 (9.1%) ^c^	44
E14.5	9 (34.6%)	17 (65.4%)	0 (0.0%)	26
P1^d^	60 (32.8%)	123 (67.2%)	0 (0.0%)	183

^a^ 3 of 57 embryos developed chest edema.

^b^ 21 of 23 embryos exhibited severe growth retardation and chest edema.

^c^ all 4 embryos were dead.

^d^ P1, postnatal day 1

### Deletion of IP_3_R genes in cardiovascular cell lineages did not cause embryonic lethality

**Fig 2:**
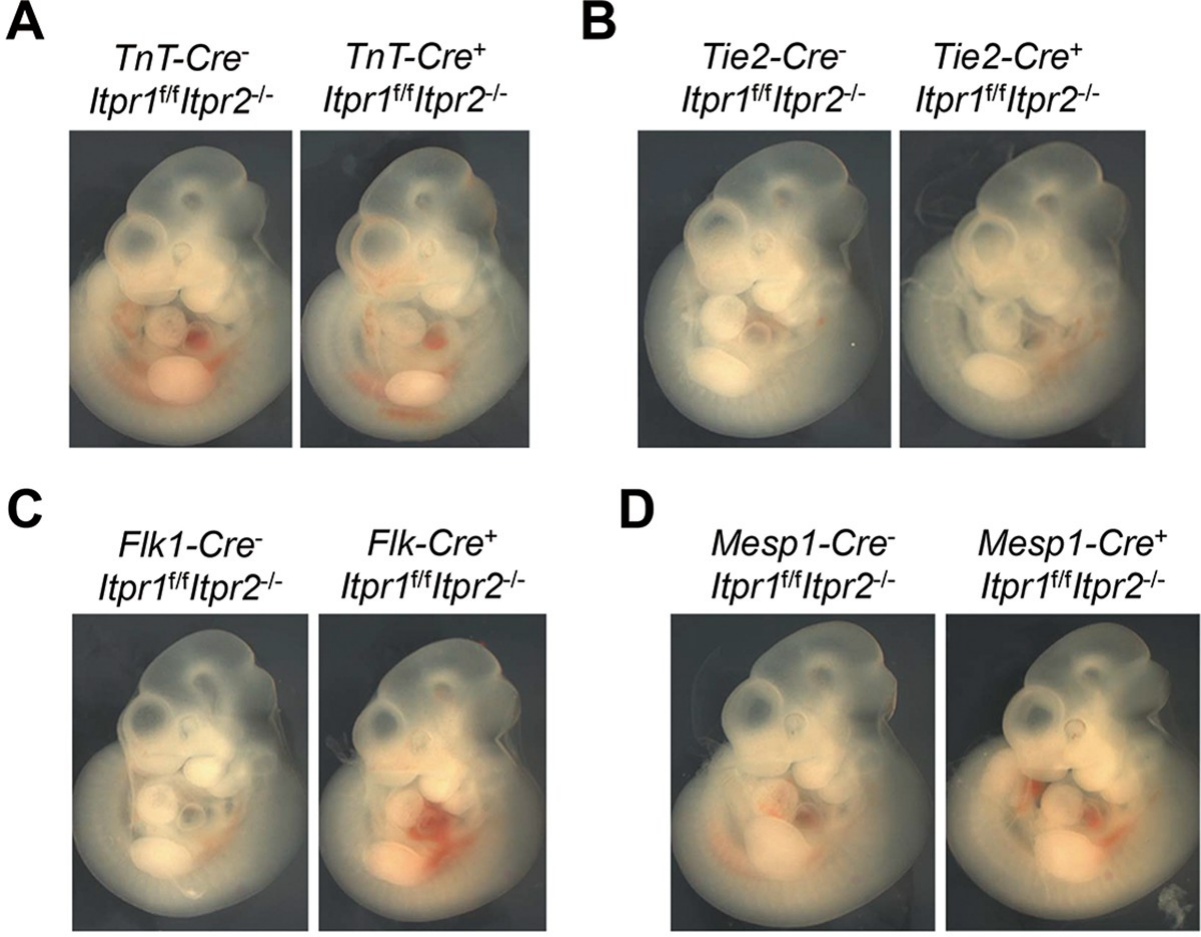
Conditional deletion of IP_3_R1 in cardiovascular cell lineages in IP_3_R2 null mice. Cardiac specific TnT-Cre (**A**), endothelial / hematopoietic cell-specific Tie2-Cre (**B**) and Flk1-Cre (**C**), and Mesp1-Cre (**D**) that targets multiple cardiovascular cell lineages were used to delete IP_3_R1 gene in IP_3_R2^-/-^ mice. Whole-mount morphological assessment of control versus conditional IP_3_R1 and IP_3_R2 double knockout mice at E10.5 revealed no growth retardation after deletion of both IP_3_R genes by TnT-Cre (n = 6), Tie2-Cre (n = 5), Flk1-Cre (n = 7), and Mesp1-Cre (n = 7).

We then investigated whether cells of cardiovascular origin were responsible for cardiovascular defects and embryonic lethality in DKO embryos. We first generated a series of conditional IP_3_R1 knockout mouse models in IP_3_R2 null background using cardiac muscle-specific expressing TnT-Cre [21], and endothelial / hematopoietic cell-specific expressing Tie2-Cre and Flk1-Cre [22,23]. It has been shown in our laboratory that TnT-Cre and Tie2-Cre are very efficient at deleting multiple floxed genes in mouse embryos [9,28,32,33]. We also performed quantitative real time PCR to examine expression of the *Itpr1* gene, and found that *Itpr1* mRNA levels were dramatically reduced in hearts of *TnT-Cre*^+^*Itpr1*^f/f^*Itpr2*^-/-^ embryos, and in blood cells of *Tie2-Cre*^+^*Itpr1*^f/f^*Itpr2*^-/-^ and *Flk1-Cre*^+^*Itpr1*^f/f^*Itpr2*^-/-^ embryos, when compared with those control embryos (**S2A–S2C Fig**). To our surprise, ablation of IP_3_R1 by TnT-Cre, Tie2-Cre or Flk1-Cre in IP_3_R2 null background did not result in any embryonic lethality (**S1 Table**). The embryos with conditional deletion of IP_3_R1 by TnT-Cre, Tie2-Cre or Flk1-Cre in IP_3_R2 null background were also phenotypically indistinguishable from control embryos at E10.5, exhibiting no growth retardation (**Fig 2A–2C**). Mesp1, a transcription factor of the b-HLH family, is the earliest marker of the cardiovascular lineages [34,35]. Cell lineage tracing analysis of Mesp1-Cre/Rosa-LacZ embryos and placentas at E9.5 have consistently shown that Mesp1-Cre can target the endothelium, endocardium, and myocardium in the embryonic heart, as well as the fetal endothelium from the allantoic capillaries in the placenta (**S3A Fig**). We also found that *Itpr1* mRNA levels in hearts of *Mesp1-Cre*^+^*Itpr1*^f/f^*Itpr2*^-/-^ mice were significantly decreased when compared with those of *Mesp1-Cre* ^-^*Itpr1*^f/f^*Itpr2*^-/-^ mice (**S2D Fig**). However, Mesp1-Cre mediated ablation of IP_3_R1 in IP_3_R2 null mice also did not cause any abnormality in embryonic development or embryonic lethality (**Fig 2D**; **S3B Fig**; **S1 Table**). Taken together, these results demonstrated that deletion of both IP_3_R1 and IP_3_R2 genes in cardiac cells, endothelial cells or even early precursors of the cardiovascular system is not responsible for early embryonic developmental defects observed in DKO mice. Therefore, our results indicate the alternative possibility that allantoic and/or placental defects may account for the embryonic lethality of DKO embryos.

### DKO embryos developed allantoic-placental defects

The placenta and allantois play an essential role in sustaining fetal growth throughout the gestational period. In mice, placental development starts at embryonic day (E) 3.5 when the outer trophectoderm layer and the inner cell mass are formed [18]. At the time of implantation (E4.5), different trophoblast cell types begin to form. The allantois arises from the mesoderm at the posterior end of the embryo and joins to the chorion at E8.5 [36]. Once the distal end of the allantois is joined with the chorion, the chorion begins to fold, making a space where fetal blood vessels grow in from the allantois to generate the fetal components of the placental vasculature. The trophoblast, together with fetal blood vessels, undergoes extensive villous branching to generate the labyrinth that is supported by the spongiotrophoblast [37,38]. The maternal blood supply passes through the spongiotrophoblast via the maternal arterial sinuses, and the umbilical artery and vein connect the fetal vasculature of the placenta to the developing fetus [38,39]. Defects in the placenta development and function are known to lead to fetal growth retardation and, in more severe cases, secondary cardiac phenotypes and embryonic lethality [38,40–42].

**Fig 3:**
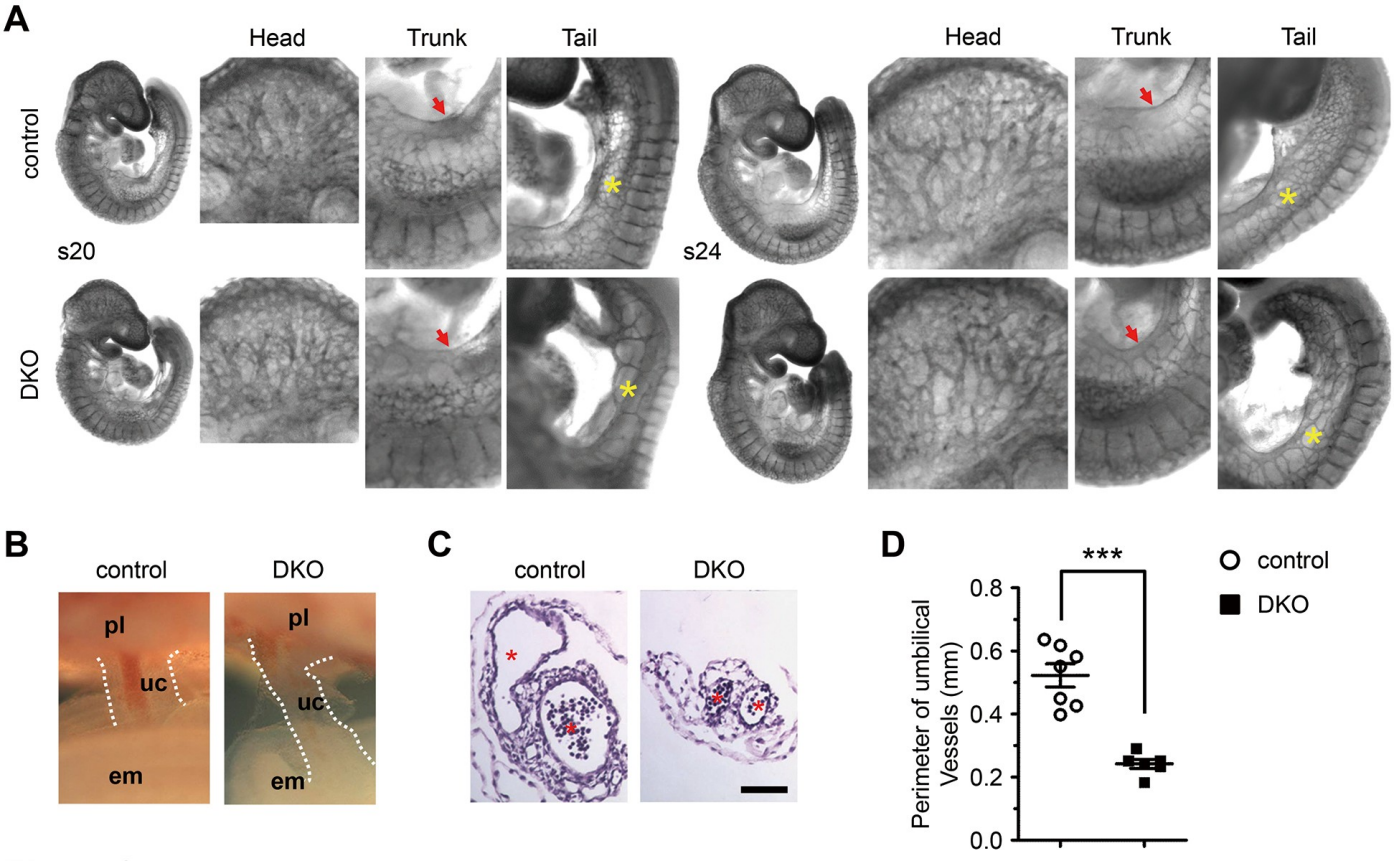
Deletion of both IP_3_R1 and IP_3_R2 resulted in developmental abnormalities in allantoic / umbilical vessels. (**A**) Whole-mount platelet endothelial cell adhesion molecule (PECAM) staining of fixed DKO and control embryos at the stages of 20 (s20) and 24 (s24) somite pairs at low and high magnifications. Please note reduced diameter of umbilical vein (red arrow) and reduced density of umbilical vein plexus (yellow star) in the caudal trunk in DKO embryos. (**B**, **C**) Whole-mount and transverse sections of umbilical cords in DKO and control mouse embryos at E9.5. The diameter of the allantois is outlined by two parallel dotted white lines. pl, placenta; em, embryo; uc, umbilical cord. The asterisks indicate the umbilical vessels. Black bar represents 70 μm. (**D**) Quantitative analysis showing that the averaged perimeter of two umbilical vessels was significantly reduced in DKO embryos (n = 6) when compared with control embryos (n = 7). All data represent mean ± SEM. Significance was determined by two-tailed, unpaired Student’s *t*-test. ***p < 0.001 versus control.

Indeed, we found that DKO embryos at E9.5 exhibited allantoic-placental abnormalities. To minimize the difference in embryonic body size, DKO and somite pair-matched control embryos with comparable growth were collected at E9.5 and examined. First of all, we found that the size/diameter of the umbilical vein that is located in the caudal trunk was significantly reduced in DKO embryos at E9.5, when compared with the somite pair-matched control embryos (**Fig 3A**; **S4A and S4B Fig**). Furthermore, the density of umbilical vein plexus was also dramatically decreased in DKO embryos, evidenced by the reduction of branches from the umbilical vein in DKO embryos when compared with control embryos (**Fig 3A**; **S4C and S4D Fig**). In addition, the size of the umbilical cord / allantois in DKO embryos was also apparently reduced at this stage (**Fig 3B**), which was further confirmed by histological assessment, evidenced by the reduction of the averaged perimeters of umbilical vessels in DKO embryos when compared with somite pair-matched control embryos (**Fig 3C** and **3D**).

**Fig 4:**
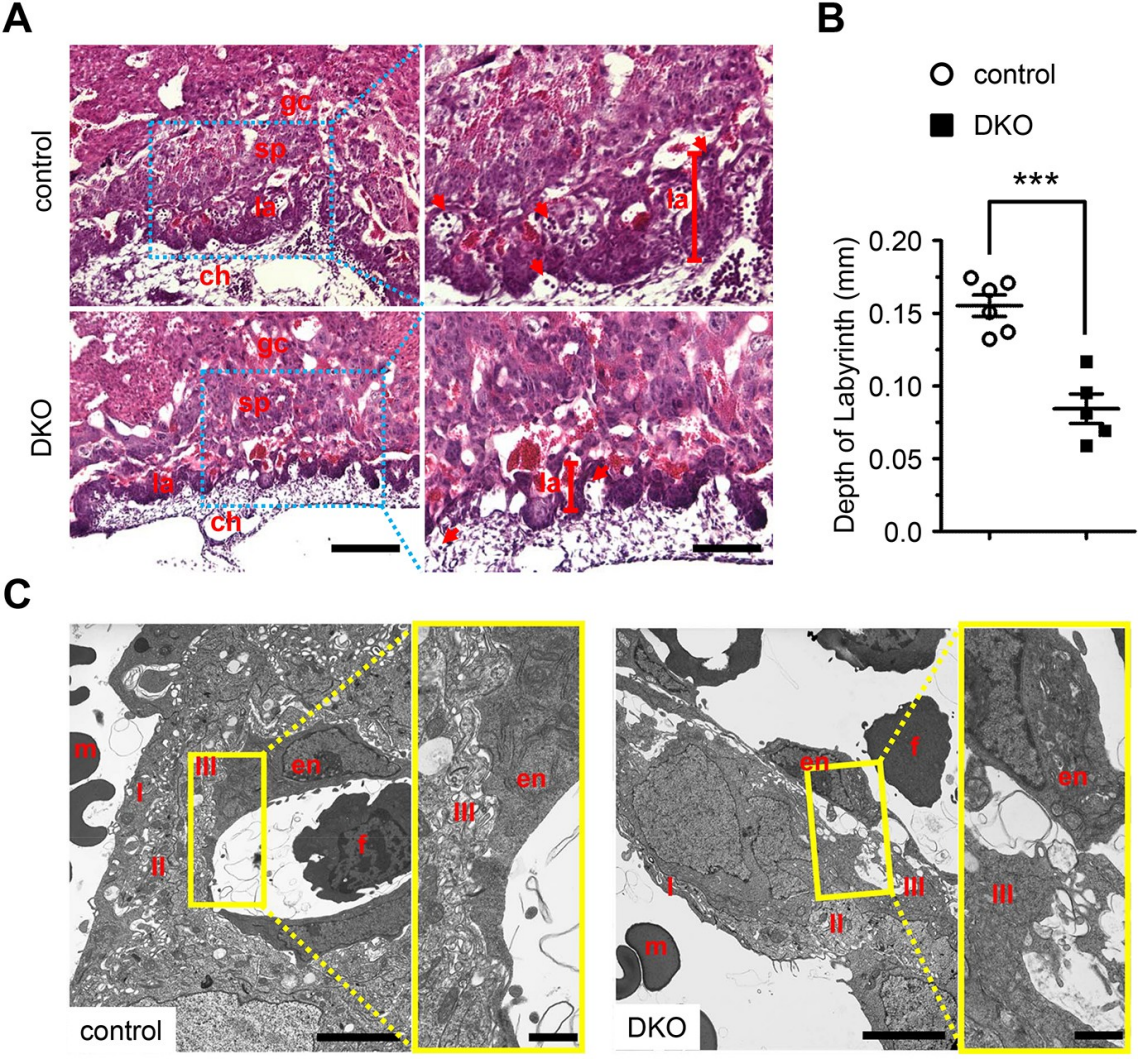
Ablation of both IP_3_R1 and IP_3_R2 caused placental defects. (**A**) Histological analysis of placental sections from E9.5 DKO and control embryos by H&E staining, at low (left) and high (right) magnification views. Trophoblast giant cell (gc), spongiotrophoblast (sp) and labyrinth (la) layers as well as chorion (ch) are shown within the placentas. The depth of the labyrinth is depicted by a vertical red bar. Red arrows denote the nucleated fetal blood cells. Black bar represents 0.2 mm (left) and 0.1 mm (right). (**B**) Quantitative analysis showing that the depth of the labyrinth is significantly reduced in DKO placentas (n = 5) when compared with control placentas (n = 6). All data represent mean ± SEM. Significance was determined by two-tailed, unpaired Student’s *t*-test. ***p < 0.001 versus control. (**C**) Ultrastructural analysis of the trophoblast barrier in E9.5 DKO and control labyrinth layer. Representative micrographs from DKO and control labyrinth layers at E9.5 at low (left) and high (right) magnification views. Fetal allantoic endothelial cells (en), maternal blood cells (m), fetal blood cells (f), the first (I), second (II), and third (III) layers of the trilaminar trophoblast layer are shown. Adjacent layers are tightly packed and intimately associated with a compacted fetal allantoic endothelium (en) in control placenta. Please note the dissociation between the fetal allantoic endothelial cells and layer III of the trilaminar trophoblast layer in the DKO placenta. Black bar represents 5 μm (left) and 1 μm (right).

We also investigated whether DKO embryos developed placental defects. For this purpose, we then assessed expression of IP_3_R subtypes in mouse placenta and allantois at E8.5–9.5. IP_3_R1 and IP_3_R3 were both expressed in cells of the maternal decidua, chorion mesoderm, spongiotrophoblast, labyrinth layer of the placenta, and the allantois (**S5A and S5C Fig**). On the other hand, assessment of IP_3_R2 expression in placenta revealed uniform expression throughout the placenta and allantois at both E8.5 to E9.5 (**S5B Fig**). Whole-mount examination of DKO embryos between E8.0-E8.5 revealed that the extension and fusion of the allantois were indistinguishable from littermate controls (**S6 Fig**), suggesting that early events of placental development were unaffected in DKO embryos. However, histological examination of placenta from DKO embryos at E9.5 revealed a strikingly underdeveloped labyrinth layer when compared to littermate controls (**Fig 4A** and **4B**; **S2 Table**). It has been shown that cell lineage derivatives from placental trophoblast and allantois mesoderm play a critical role in the establishment and maturation of the labyrinth layer [40,42]. Therefore, we next determined whether trophoblast cell specific markers, such as the basic helix-loop-helix transcription factor *Hand1*, chorionic somatomammotropin hormone 1 (*Csh1*) and the mammalian Distal-less homolog *Dlx3* [43–45], were affected in DKO placentas. Expression of *Hand1*, *Csh1*, and *Dlx3* could be identified in both DKO and control placentas (**S7 Fig**), suggesting that trophoblast cell identify was unaffected after IP_3_R deficiency. However, expression of *Dlx3*, a specific marker of the trilaminar trophoblast layer, was much more restricted in DKO placentas compared to the controls (**S7 Fig**), which further highlights the labyrinth defect observed in DKO placentas.

In addition, ultrastructural analysis of DKO placentas at E9.5 revealed the dissociation between fetal allantoic endothelium and syncytiotrophoblast layer III of the trilaminar trophoblast layer (**Fig 4C**), which was in contrast to the littermate controls, which exhibited an intimate association between the compacted fetal allantoic endothelium and the trilaminar trophoblast layer (**Fig 4C**), suggesting that deletion of both IP_3_R1 and IP_3_R2 in mice is required for maintaining the chorio-allantoic integrity.

### Epiblast-specific deletion of IP_3_R1 in IP_3_R2 null background results in embryonic lethality and placental defects

**Fig 5:**
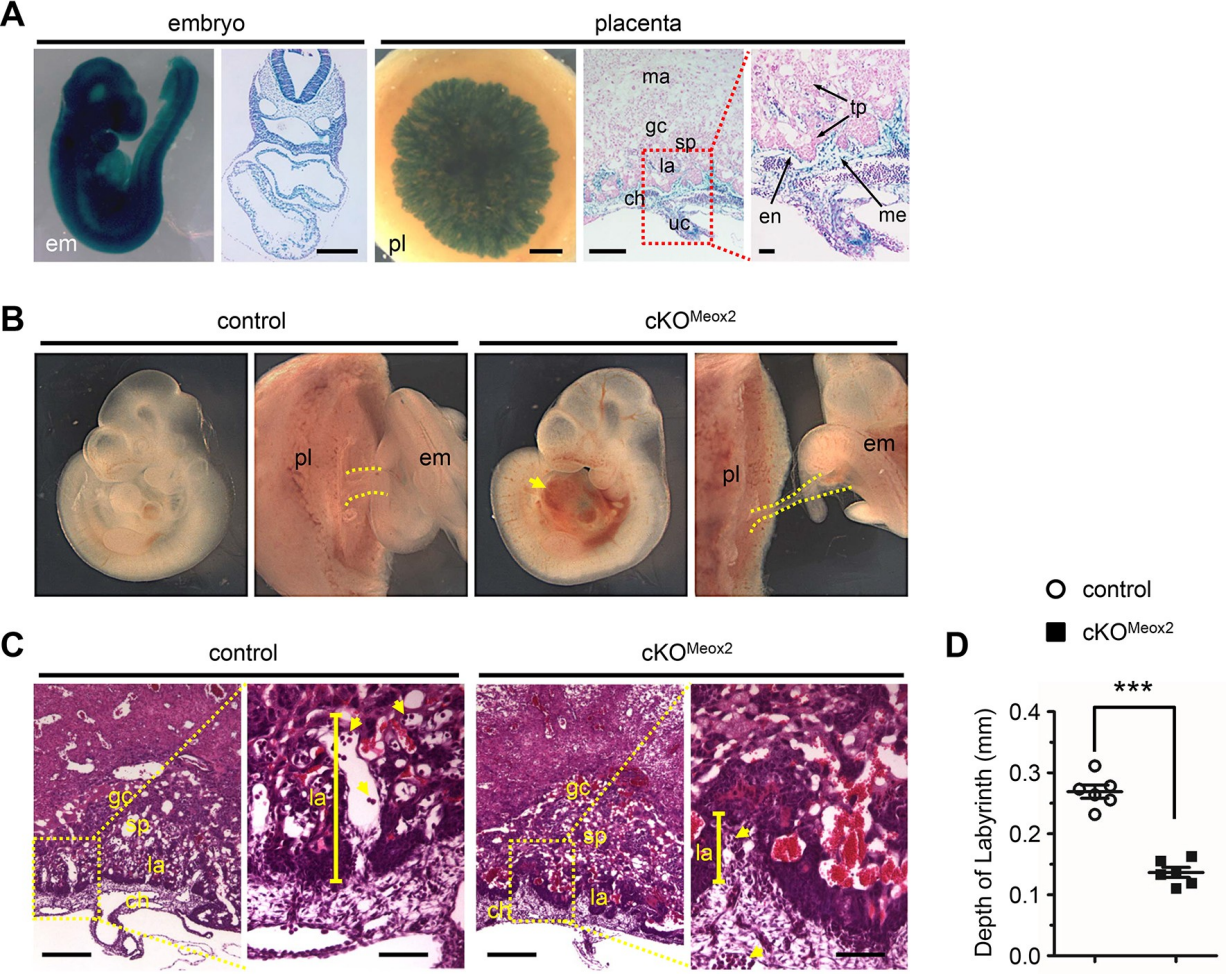
IP_3_R1 and IP_3_R2 deletion in the epiblast causes placental defects. (**A**) Whole mount lacZ staining and transverse sections counterstained with nuclear fast red in E9.5 Meox2-Cre/Rosa-lacZ embryos and placentas. Low and high magnification views of the umbilical cord were presented to highlight the contribution of Meox2-Cre derived cells in the umbilical cord and placenta. em, embryo; pl, placenta; uc, umbilical cord; ma, maternal decidua; ch, chorion; gc, trophoblast giant cell; sp, spongiotrophoblast; la, labyrinth; en, endothelial cell; tp, trophoblast cell; me, mesenchymal cell. Black bar represents 0.4 mm. (**B**) Whole-mount assessment of E10.5 control and *Meox2-Cre*^+^*Itpr1*^f/f^*Iptr2*^-/-^ (cKO^Meox2^) embryos. The diameter of the umbilical cord is outlined by two parallel dotted yellow lines. Please note chest edema (yellow arrow) in cKO^Meox2^ embryos. (**C**) Histological analysis of placental sections from E10.5 cKO^Meox2^ and control embryos by H&E staining, at low (left) and high (right) magnification views. Trophoblast giant cell (gc), spongiotrophoblast (sp) and labyrinth (la) layers as well as chorion (ch) are shown within the placentas. The depth of the labyrinth is depicted by a vertical yellow bar. Yellow arrows denote the nucleated fetal blood cells. Black bar represents 0.4 mm (left) and 0.1 mm (right). (**D**) Quantitative analysis showing that the depth of the labyrinth is significantly reduced in E10.5 cKO^Meox2^ placentas (n = 6) when compared with control placentas (n = 6). All data represent mean ± SEM. Significance was determined by two-tailed, unpaired Student’s *t*-test. ***p < 0.001 versus control.

We next determined whether IP_3_Rs within cells from epiblast lineages, which give rise to the allantois, chorionic mesoderm, and embryo [46–48], were required for embryonic viability. We thus ablated IP_3_R1 in IP_3_R2 null background utilizing Meox2-Cre, which is active in cells of the epiblast, but is not active in primitive endoderm or trophectoderm [25]. Consistently, cell lineage tracing analysis at E9.5 Meox2-Cre/Rosa-LacZ embryos and placentas revealed that Meox2-Cre activity was highly efficient in all cells of E9.5 embryos including extraembryonic vascular and mesenchymal cells, and chorionic mesoderm, but not in extraembryonic trophoblast cells (**Fig 5A**). It is important to note that Meox2-Cre mediated excision of IP_3_R1 in IP_3_R2 null (cKO^Meox2^) mice also resulted in similar but slightly late-onset allantoic-placental phenotypes and embryonic lethality (**Fig 5B**; **Table 2**), when compared with global DKO embryos. At E10.5, cKO^Meox2^ embryos also exhibited a dramatic decrease in the diameter of the umbilical cord (**Fig 5B**). Furthermore, an underdeveloped labyrinth layer was also observed in placentas of cKO^Meox2^ embryos at E10.5 when compared to the littermate controls (**Fig 5C** and **5D**). These results all suggested that epiblast cell lineages could, at least partially, account for the phenotype of global IP_3_R1 and IP_3_R2 double knockout embryos.

**Table 2:** Genotypic analysis of embryos from *Meox2-Cre*^+^*Itpr1*^f/+^*Itpr2*^-/-^ x *Itpr1*^f/f^*Itpr2*^-/-^ intercrosses.

Day of analysis	No. of genotype				Total
	*Meox2-Cre*^-^ *Itpr1*^f/+^*Itpr2*^-/-^	*Meox2-Cre*^-^ *Itpr1*^f/f^*Itpr2*^-/-^	*Meox2-Cre*^+^ *Itpr1*^f/+^*Itpr2*^-/-^	*Meox2-Cre*^+^ *Itpr1*^f/f^*Itpr2*^-/-^	
E8.5	7 (18.9%)	11 (29.7%)	9 (24.3%)	10 (27.0%)	37
E9.5	12 (28.6%)	9 (21.4%)	10 (23.8%)	11 (26.2%)	42
E10.5	13 (27.7%)	15 (31.9%)	10 (21.3%)	9 (19.1%) ^a^	47
E11.5	9 (30.0%)	8 (26.7%)	6 (20.0%)	7 (23.3%) ^b^	30
E12.5	8 (25.0%)	9 (28.1%)	10 (31.3%)	5 (15.6%) ^c^	32
E14.5	6 (35.3%)	4 (23.5%)	7 (41.2%)	0 (0.0%)	17
P1^d^	35 (30.2%)	40 (34.5%)	41 (35.3%)	0 (0.0%)	116

^a^ 9 of 9 embryos developed thin umbilical vessels; 4 of 9 embryos developed chest edema.

^b^ 7 of 7 embryos exhibited severe growth retardation and chest edema.

^c^ 5 of 5 embryos were dead.

^d^ P1, postnatal day 1

## Discussion

In this study, we investigated the role of IP_3_R1 and IP_3_R2 in embryonic development and survival using both conventional and tissue-specific gene knockout strategies. We demonstrated that ablation of both IP_3_R1 and IP_3_R2 resulted in growth retardation and embryonic lethality at around E10.5. A previous study also showed that deletion of both IP_3_R1 and IP_3_R2 caused death of mutant embryos around E10.5 [6]; in a subset of E9.5 DKO embryos which had already developed obvious growth retardation, we observed similar cardiac defects as previously reported [6], including enlarged ventricles and thinner ventricular walls. Our study also revealed no obvious cardiac defects in DKO embryos that did not develop obvious growth retardation at E9.5. Furthermore, our results also demonstrated that cardiac cell-specific deletion of IP_3_R1 in IP_3_R2 null background genes did not cause any growth retardation and embryonic lethality, suggesting that IP_3_R1 and IP_3_R2 in cardiac cells are dispensable for embryonic development, which also implicated that cardiac defects observed in DKO embryos might be secondary to the allantoic-placental defects observed in DKO embryos.

On the other hand, placental defects strongly correlate with abnormal heart, brain and vascular development, even though the mechanistic relationship between these systems has not been fully understood [17,49]. In our study, allantoic-placental defects could be detected in all DKO embryos, even those without obvious growth retardation, implicating that allantoic-placental defects could precede cardiovascular abnormalities in DKO embryos. These allantoic-placental defects observed in DKO embryos included (1) reduced size of umbilical veins and less umbilical vein plexus in the posterior trunk, (2) smaller umbilical cord and reduced diameter of both umbilical vein and artery, (3) underdeveloped labyrinth layer in the placenta, and (4) diminished connection between endothelium of fetal allantoic capillary and the trilaminar trophoblast layer. All these abnormalities in DKO embryos could lead to insufficient metabolic exchange and eventually embryonic lethality.

It has been suggested that Ca^2+^ signaling pathways might be involved in regulating the establishment of fetal-maternal connection. PLCδ1/PLCδ3 double-knockout mice exhibited decreased vascularization in the labyrinth layer of the placenta and abnormal proliferation and apoptosis of trophoblasts [50]. Na^+^-Ca^2+^ exchanger 1 (NCX1), a key plasma membrane Ca^2+^ transporter, was also involved in both cardiac and placental development. NCX1 knockout mice developed both cardiac defects and an underdeveloped labyrinth layer [51,52], but it is important to note that cardiac specific-expression of NCX1 in NCX1 knockout mice could only rescue cardiac defects, not placental defects [53]. Furthermore, the study by Uchida et al [7], also showed that double knockout of IP_3_R1 and IP_3_R3 caused embryonic lethality at a similar stage to that of our IP_3_R1 and IP_3_R2 double knockout mice. Uchida et al also observed that deletion of IP_3_R1 and IP_3_R3 resulted in abnormal vascular development in the allantois and disorganized placenta. Although their results suggested that inhibition of IP_3_Rs in cultured endothelial cells could affect tube formation and cell migration, they did not generate endothelial cell-specific IP_3_R1 and IP_3_R3 knockout mice for further characterization. Our results showing no phenotype in mice with deletion of IP_3_R1 by Tie2-Cre and Flk1-Cre in IP_3_R2 null background make it unlikely that loss of IP_3_R1 and IP_3_R2 in endothelial cells could account for vascular defects observed in the global knockout embryos.

In our present study, we utilized the cell / tissue specific gene deletion strategy to investigate the cell dependent mechanism underlying how IP_3_R1 and IP_3_R2 regulate the allantoic-placental development. Firstly, endothelial / hematopoietic cell-specific deletion of IP_3_R1 in IP_3_R2 null background did not cause similar developmental defects and embryonic lethality in mice. Secondly, ablation of IP_3_R1 in IP_3_R2 null background by Mesp1-Cre, which is mainly expressed in anterior mesoderm targeting multiple cardiovascular lineages [34,35], also did not cause embryonic developmental defect and embryonic death. These results together strongly suggested that vascular abnormalities observed in DKO embryos might be secondary to the allantoic / placental defects. Indeed, expression of all three IP_3_R subtypes could be found in the placenta, including the allantois, chorion mesoderm, and trophoblast cells. Trophoblast cells are derived from the trophectoderm that constitutes the outer layer of the blastocyst and segregates from the inner cell mass [54], and defects in trophoblast differentiation and function have been shown to cause embryonic lethality [45,55,56]. On the other hand, the inner cell mass gives rise to primitive endoderm (hypoblast) and primitive ectoderm (epiblast), and the latter forms all the fetal tissues, both somatic and germline, as well as the amnion ectoderm and all the extraembryonic mesoderm [46,47]. It is important to note that epiblast-specific deletion of IP_3_R1 by Meox2-Cre in IP_3_R2 null background also resulted in similar allantoic-placental defects in mice, even including the slight delay in embryonic death compared to conventional global DKO embryos. Although it remains unclear whether deletion of IP_3_Rs in trophoblast cells could also contribute to the developmental abnormalities observed in DKO embryos, our results have at least demonstrated that IP_3_R1 and IP_3_R2 in epiblast cell lineages are required for normal embryonic development and survival. It is worthy to note that deletion of IP_3_R1 by Meox2-Cre but not Mesp1-Cre in IP_3_R2 null background could cause allantoic-placental defects and embryonic lethality. Mesp1-Cre activity has been shown to partially overlap with that of Meox2-Cre [24,25,35], but our cell lineage tracing also revealed that the latter could target both allantois-derived extraembryonic mesenchymal cells and chorionic mesoderm more efficiently. In this context, it will be very interesting, in future studies, to investigate whether IP_3_Rs in allantois-derived cells or chorionic mesoderm could account for the allantoic-placental defects in the DKO embryos.

## Supporting information

S1 Fig Expression of IP_3_R subtypes in mouse embryos at early developmental stages. Whole-mount RNA in situ hybridization was used to identify expression of *Itpr1*, *Itpr2* and *Itpr3* in mouse embryos from E8.5–11.0 (**A**-**O**). pa, pharyngeal arch artery; sv, sinus venosus; v, ventricle; avc, atrioventricular cushion; rv, right ventricle; ra, right atria; lv, left ventricle; ot, outflow tract; la, left atria; fb, forelimb bud. The angled yellow line denotes the position of the section used to detect IP_3_R mRNA expression in the heart at a low (**D**, **I**, **N**) magnification view. A high (**E**, **J**, **O**) magnification view depicts the dotted outlined region in D, I, and N, and demonstrates IP_3_R mRNA expression in the endocardium of the heart. Black bar represents 0.25 mm (D, I, N) and 0.10 mm (E, J, O).
(TIF)

S2 Fig Characterization of *Itpr1* mRNA levels in conditional gene knockout mouse models. Quantitative real time PCR was used to measure the mRNA levels of IP_3_R1 in hearts of *TnT-Cre*^+^*Itpr1*^f/f^*Itpr2*^-/-^ embryos (**A**; n = 4), in blood cells of *Tie2-Cre*^+^*Itpr1*^f/f^*Itpr2*^-/-^ (**B**; n = 3) and *Flk1-Cre*^+^*Itpr1*^f/f^*Itpr2*^-/-^ (**C**; n = 4) embryos, and in hearts of *Mesp1-Cre*^+^*Itpr1*^f/f^*Itpr2*^-/-^ (**D**; n = 3) embryos at E10.5. The tissues from *TnT-Cre*^-^*Itpr1*^f/f^*Itpr2*^-/-^ (n = 4), *Tie2-Cre*^-^*Itpr1*^f/f^*Itpr2*^-/-^ (n = 3), *Flk1-Cre*^-^*Itpr1*^f/f^*Itpr2*^-/-^ (n = 4), and *Mesp1-Cre*^+^*Itpr1*^f/f^*Itpr2*^-/-^ (n = 3) embryos were used as control. All data represent mean ± SEM. Significance was determined by performing a two-tailed, unpaired Student’s *t*-test. *p < 0.05, **p < 0.01 versus control.
(TIF)

S3 Fig Deletion of IP_3_R1 by Mesp1-Cre in IP_3_R2 null background does not alter embryonic development. (**A**) Whole mount lacZ staining and transverse sections counterstained with nuclear fast red in E9.5 Mesp1-Cre/Rosa-lacZ embryos and placentas. Low and high magnification views of the umbilical cord were presented to highlight the contribution of Mesp1-Cre derived cells in the umbilical cord and placenta. em, embryo; pl, placenta; uc, umbilical cord; ma, maternal decidua; ch, chorion; gc, trophoblast giant cell; sp, spongiotrophoblast; la, labyrinth; en, endothelial cell; tp, trophoblast cell; me, mesenchymal cell. Black bar represents 0.4 mm. (**B**) Whole-mount assessment of *Mesp1-Cre*^-^*Itpr1*^f/f^*Iptr2*^-/-^ and *Mesp1-Cre*^+^*Itpr1*^f/f^*Iptr2*^-/-^ embryos at E10.5. Please note normal umbilical cords / vessels (yellow arrows) in *Mesp1-Cre*^+^*Itpr1*^f/f^*Iptr2*^-/-^ embryos.
(TIF)

S4 Fig Quantitative analysis of vascular development in control and DKO embryos. (**A** and **B**) The diameter of the umbilical vein that is located in the caudal trunk was measured in control and DKO embryos at the 20 somite (**A**; control, n = 3; DKO, n = 3) and 24 somite (**B**; control, n = 4; DKO, n = 3) stages, respectively. (**C** and **D**) The numbers of branches from the umbilical vein were also measured in both control and DKO embryos at the 20 somite (**C**; control, n = 3; DKO, n = 3) and 24 somite (**D**; control, n = 4; DKO, n = 3) stages, respectively. All data represent mean ± SEM. Significance was determined by performing a two-tailed, unpaired Student’s *t*-test. *p < 0.05, **p < 0.01 versus control.
(TIF)

S5 Fig Expression of IP_3_R subtypes in mouse placentas at early developmental stages. RNA in situ hybridization was used to identify expression of *Itpr1* (**A**), *Itpr2* (**B**), and *Itpr3* (**C**) in cryosections of control mouse placenta at E8.5 and E9.5. The upper right inset depicts the high magnification view of the red-dotted region. ma, maternal decidua; ch, chorion; la, labyrinth; sp, spongiotrophoblast; em, embryo; al, allantois. Black bars represent 0.4 mm at E9.5 and 0.2 mm at E8.5, respectively.
(TIF)

S6 Fig Macroscopic assessment of allantois extension and fusion in DKO mouse embryos. Whole mount assessment of DKO embryos and littermate controls at E8.25 (**A**, **B**) and E8.5 (**C**, **D**). Red arrows indicate the allantois.
(TIF)

S7 Fig Expression of trophoblast cell-specific marker in control and DKO placentas. RNA In situ hybridization was used to identify expression of *Hand1*, *Csh1*, and *Dlx3* in control and DKO placentas. Please note the restricted expression of *Dlx3* in the DKO placentas when compared with control placentas. Black bars represent 0.4 mm.
(TIF)

S1 Table Genotypic analysis of embryos for generation of cell / tissue specific IP_3_R1 and IP_3_R2 knockout mice. To generate cell / tissue specific IP_3_R1 and IP_3_R2 double knockout mice, male Cre^+^*Itpr1*^f/+^*Itpr2*^-/-^ mice were crossed with female Cre^-^*Itpr1*^f/f^*Itpr2*^-/-^ mice. The genotypes were first analyzed at postnatal day 1 (P1) to see whether the offspring were born at Mendelian ratios, and the embryos at E10.5 were then collected for morphological analysis.
(DOCX)

S2 Table Summary of embryonic phenotypes observed in IP_3_R1 and IP_3_R2 double knockout embryos between E9.5 and E11.5. ^a^ Not investigated.
(DOCX)
