# Supplementary figures and images for: Inositol 1,4,5-trisphosphate receptors are essential for fetal-maternal connection and embryo viability
Source: PLoS Genet. 2020 Apr 22;16(4):e1008739. doi: 10.1371/journal.pgen.1008739 (PMC7176088; doi:10.1371/journal.pgen.1008739)

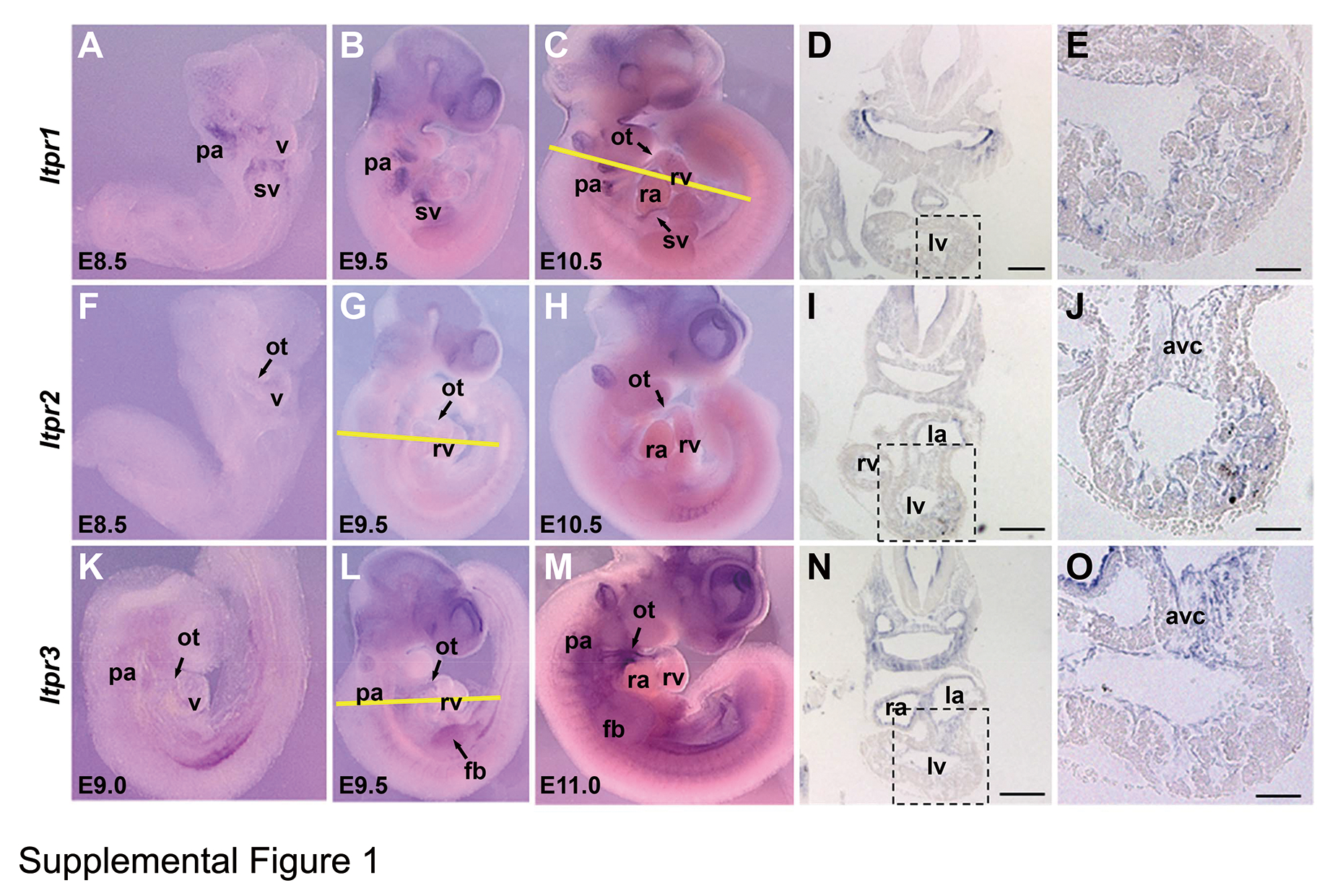

Supplement: S1 Fig — Whole-mount RNA in situ hybridization was used to identify expression of Itpr1, Itpr2 and Itpr3 in mouse embryos from E8.5–11.0 (A-O). pa, pharyngeal arch artery; sv, sinus venosus; v, ventricle; avc, atrioventricular cushion; rv, right ventricle; ra, right atria; lv, left ventricle; ot, outflow tract; la, left atria; fb, forelimb bud. The angled yellow line denotes the position of the section used to detect IP3R mRNA expression in the heart at a low (D, I, N) magnification view. A high (E, J, O) magnification view depicts the dotted outlined region in D, I, and N, and demonstrates IP3R mRNA expression in the endocardium of the heart. Black bar represents 0.25 mm (D, I, N) and 0.10 mm (E, J, O). (TIF) [file pgen.1008739.s001.tif]

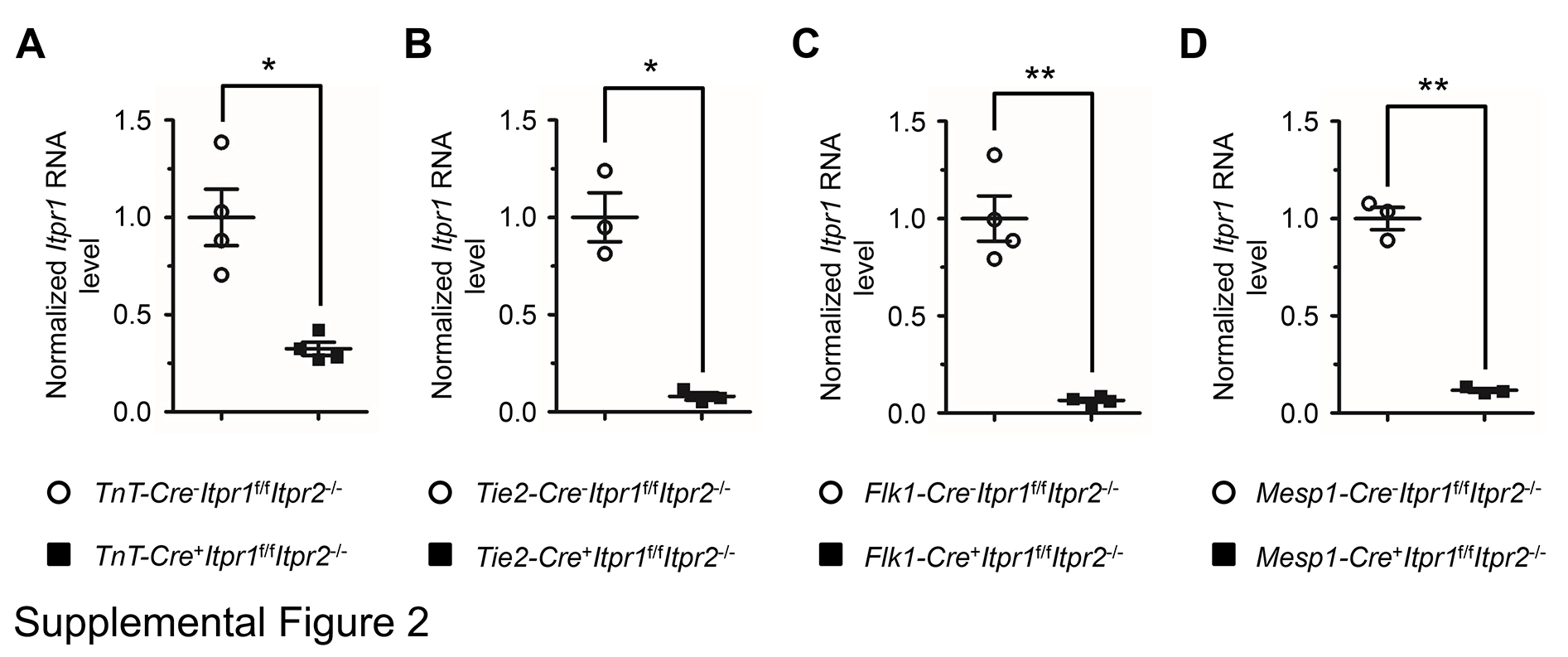

Supplement: S2 Fig — Quantitative real time PCR was used to measure the mRNA levels of IP3R1 in hearts of TnT-Cre+Itpr1f/fItpr2-/- embryos (A; n = 4), in blood cells of Tie2-Cre+Itpr1f/fItpr2-/- (B; n = 3) and Flk1-Cre+Itpr1f/fItpr2-/- (C; n = 4) embryos, and in hearts of Mesp1-Cre+Itpr1f/fItpr2-/- (D; n = 3) embryos at E10.5. The tissues from TnT-Cre-Itpr1f/fItpr2-/- (n = 4), Tie2-Cre-Itpr1f/fItpr2-/- (n = 3), Flk1-Cre-Itpr1f/fItpr2-/- (n = 4), and Mesp1-Cre+Itpr1f/fItpr2-/- (n = 3) embryos were used as control. All data represent mean ± SEM. Significance was determined by performing a two-tailed, unpaired Student’s t-test. *p < 0.05, **p < 0.01 versus control. (TIF) [file pgen.1008739.s002.tif]

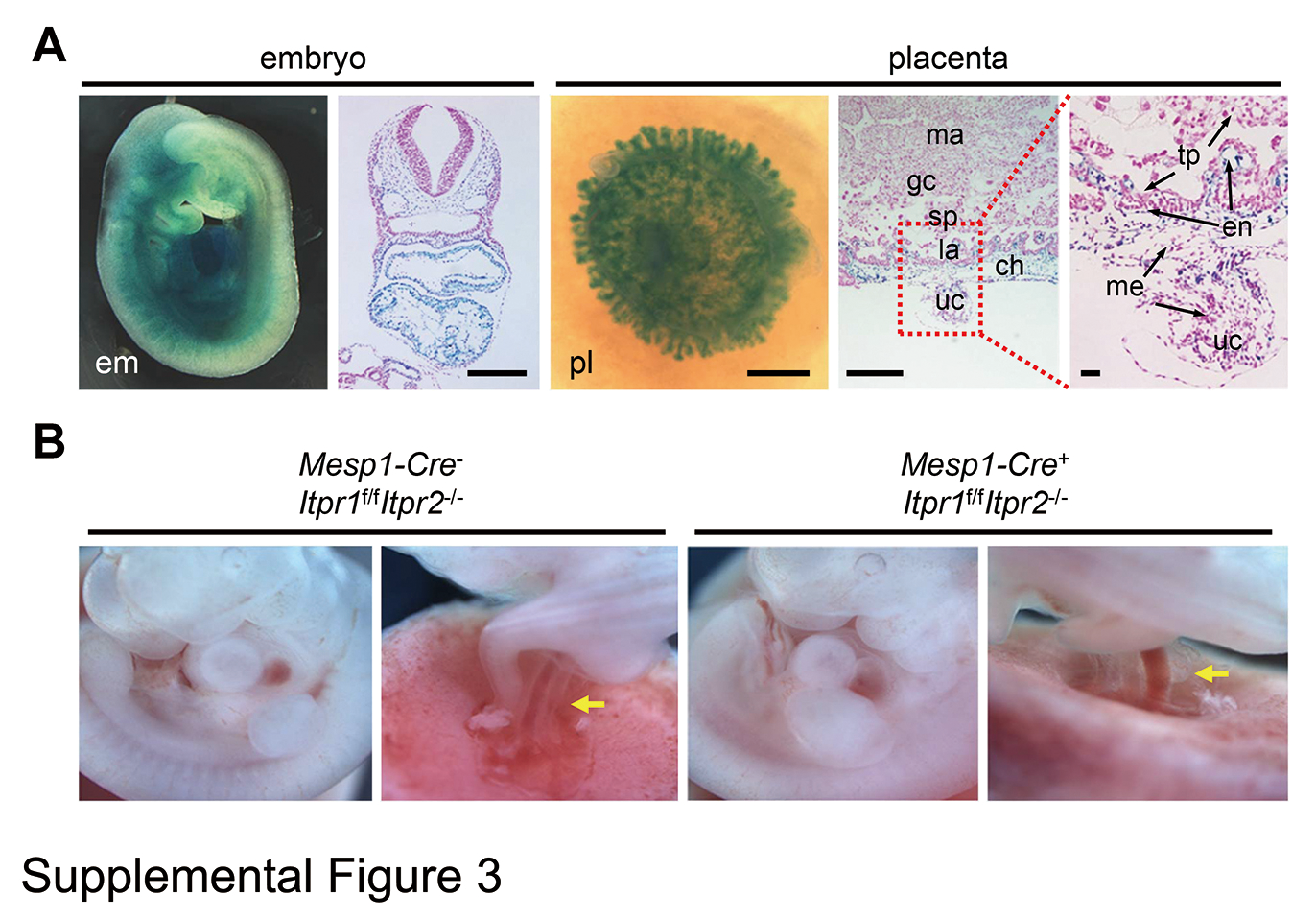

Supplement: S3 Fig — (A) Whole mount lacZ staining and transverse sections counterstained with nuclear fast red in E9.5 Mesp1-Cre/Rosa-lacZ embryos and placentas. Low and high magnification views of the umbilical cord were presented to highlight the contribution of Mesp1-Cre derived cells in the umbilical cord and placenta. em, embryo; pl, placenta; uc, umbilical cord; ma, maternal decidua; ch, chorion; gc, trophoblast giant cell; sp, spongiotrophoblast; la, labyrinth; en, endothelial cell; tp, trophoblast cell; me, mesenchymal cell. Black bar represents 0.4 mm. (B) Whole-mount assessment of Mesp1-Cre-Itpr1f/fIptr2-/- and Mesp1-Cre+Itpr1f/fIptr2-/- embryos at E10.5. Please note normal umbilical cords / vessels (yellow arrows) in Mesp1-Cre+Itpr1f/fIptr2-/- embryos. (TIF) [file pgen.1008739.s003.tif]

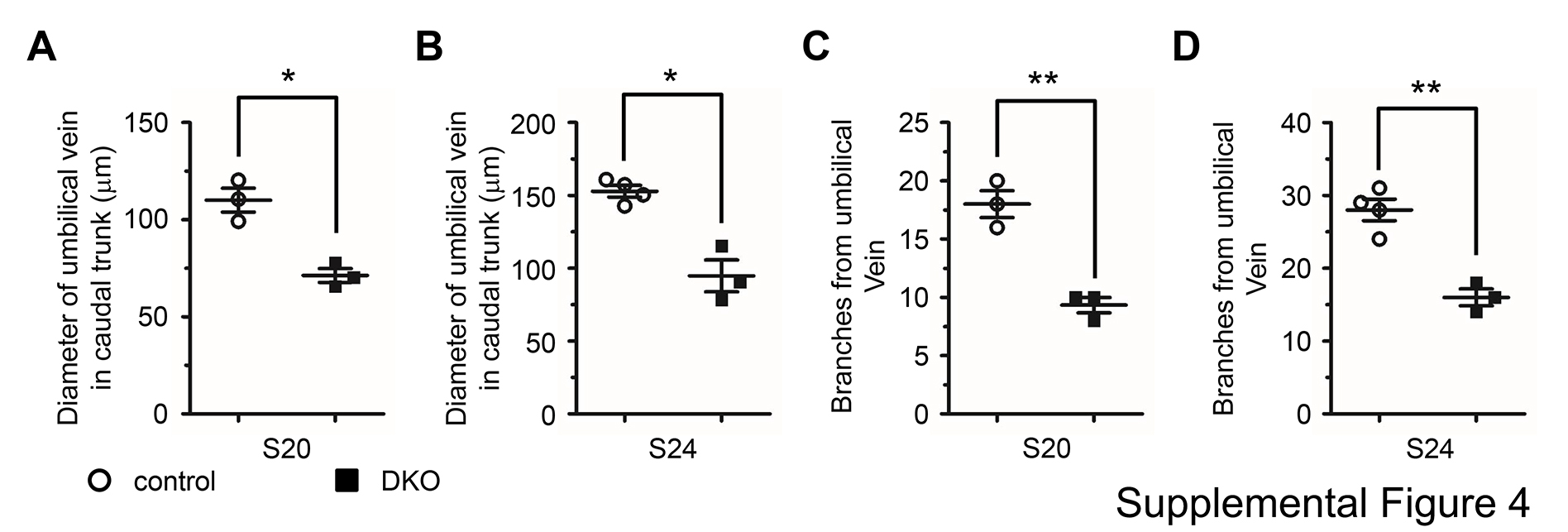

Supplement: S4 Fig — (A and B) The diameter of the umbilical vein that is located in the caudal trunk was measured in control and DKO embryos at the 20 somite (A; control, n = 3; DKO, n = 3) and 24 somite (B; control, n = 4; DKO, n = 3) stages, respectively. (C and D) The numbers of branches from the umbilical vein were also measured in both control and DKO embryos at the 20 somite (C; control, n = 3; DKO, n = 3) and 24 somite (D; control, n = 4; DKO, n = 3) stages, respectively. All data represent mean ± SEM. Significance was determined by performing a two-tailed, unpaired Student’s t-test. *p < 0.05, **p < 0.01 versus control. (TIF) [file pgen.1008739.s004.tif]

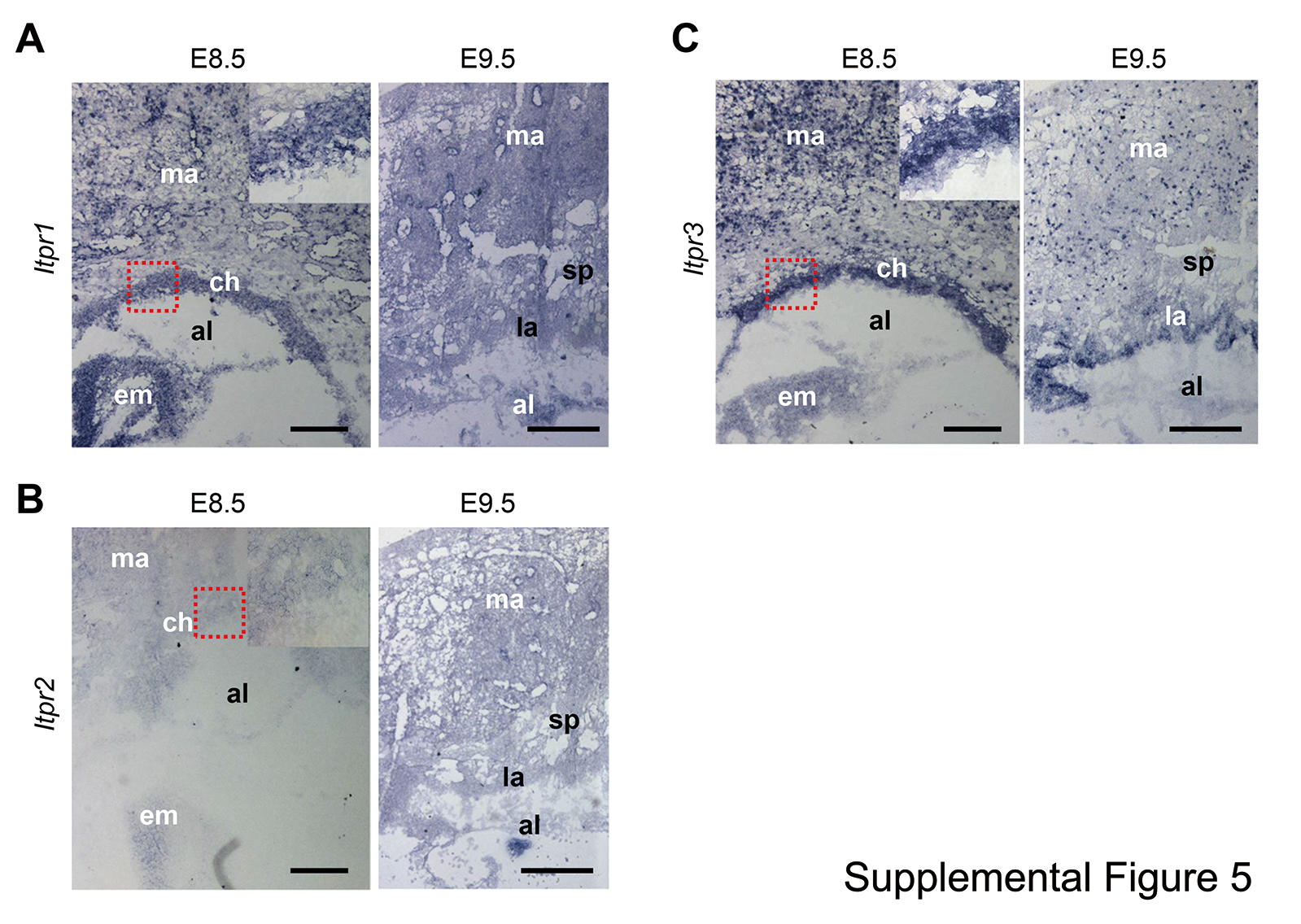

Supplement: S5 Fig — RNA in situ hybridization was used to identify expression of Itpr1 (A), Itpr2 (B), and Itpr3 (C) in cryosections of control mouse placenta at E8.5 and E9.5. The upper right inset depicts the high magnification view of the red-dotted region. ma, maternal decidua; ch, chorion; la, labyrinth; sp, spongiotrophoblast; em, embryo; al, allantois. Black bars represent 0.4 mm at E9.5 and 0.2 mm at E8.5, respectively. (TIF) [file pgen.1008739.s005.tif]

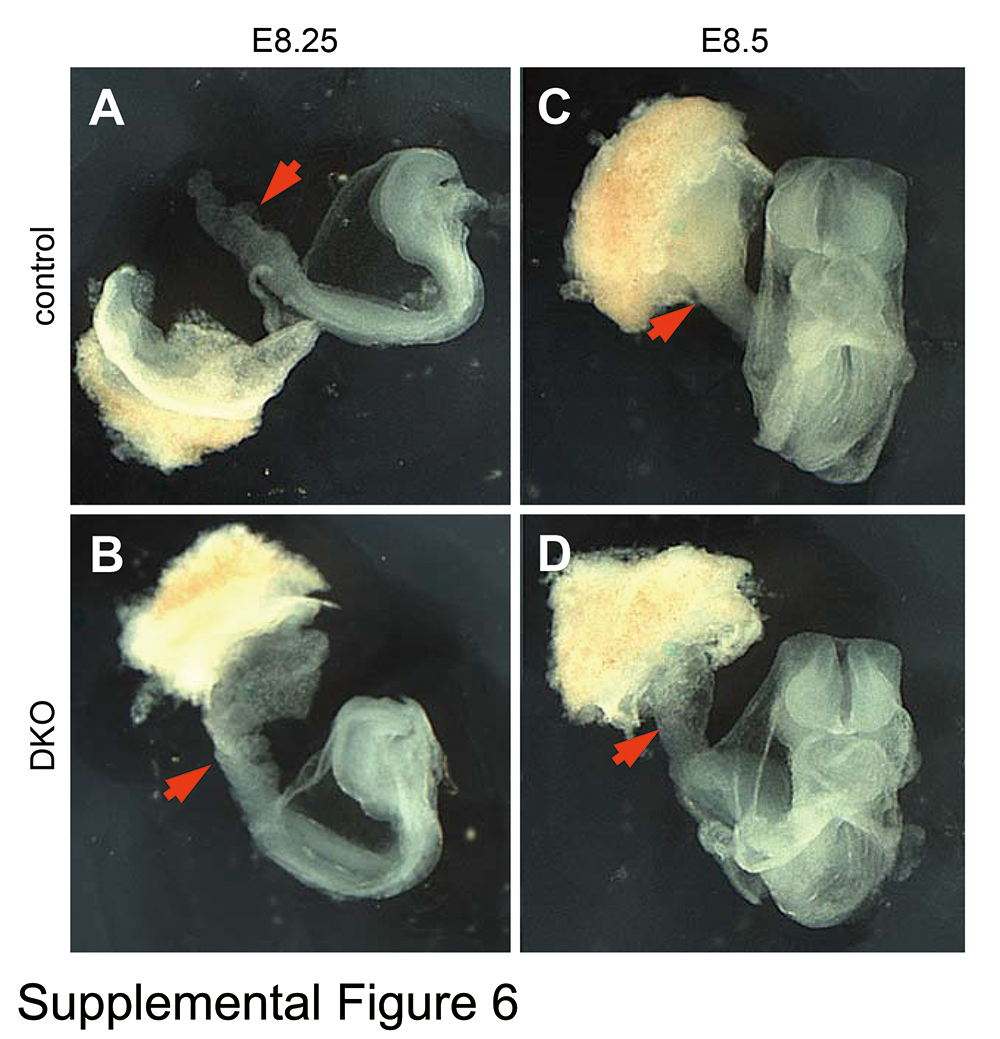

Supplement: S6 Fig — Whole mount assessment of DKO embryos and littermate controls at E8.25 (A, B) and E8.5 (C, D). Red arrows indicate the allantois. (TIF) [file pgen.1008739.s006.tif]

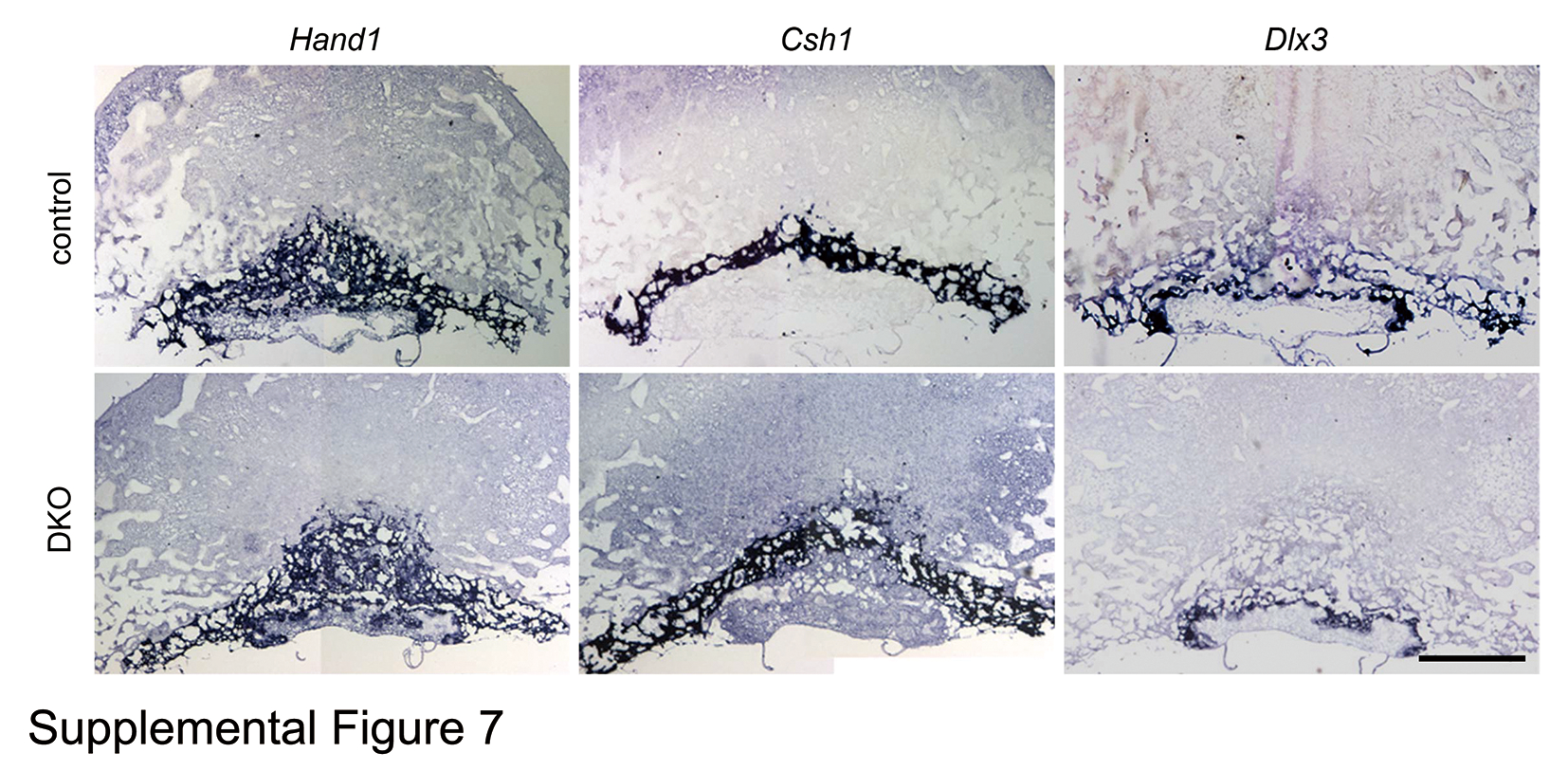

Supplement: S7 Fig — RNA In situ hybridization was used to identify expression of Hand1, Csh1, and Dlx3 in control and DKO placentas. Please note the restricted expression of Dlx3 in the DKO placentas when compared with control placentas. Black bars represent 0.4 mm. (TIF) [file pgen.1008739.s007.tif]
