# Supplementary material for: Inositol 1,4,5-trisphosphate receptors are essential for fetal-maternal connection and embryo viability
Source: PLoS Genet. 2020 Apr 22;16(4):e1008739. doi: 10.1371/journal.pgen.1008739 (PMC7176088; doi:10.1371/journal.pgen.1008739)
Supplement: S1 Table — To generate cell / tissue specific IP3R1 and IP3R2 double knockout mice, male Cre+Itpr1f/+Itpr2-/- mice were crossed with female Cre-Itpr1f/fItpr2-/- mice. The genotypes were first analyzed at postnatal day 1 (P1) to see whether the offspring were born at Mendelian ratios, and the embryos at E10.5 were then collected for morphological analysis. (DOCX) [file pgen.1008739.s008.docx]

| Type of Cre | Day of analysis | No. of genotype | | | | Total |
| --- | --- | --- | --- | --- | --- | --- |
|  |  | *Cre*^-^  *Itpr1*^f/+^*Itpr2*^-/-^ | *Cre*^-^  *Itpr1*^f/f^*Itpr2*^-/-^ | *Cre*^+^  *Itpr1*^f/+^*Itpr2*^-/-^ | *Cre*^+^  *Itpr1*^f/f^*Itpr2*^-/-^ |  |
| TnT-Cre | E10.5 | 6 (22.2%) | 5 (18.5%) | 10 (37.0%) | 6 (22.2%) | 27 |
|  | P1 | 25 (19.2%) | 38 (29.2%) | 35 (26.9%) | 32 (24.6%) | 130 |
| Tie2-Cre | E10.5 | 4 (19.0%) | 7 (33.3%) | 5 (23.8%) | 5 (23.8 %) | 21 |
|  | P1 | 30 (25.4%) | 33 (27.9%) | 28 (23.7%) | 27 (22.8%) | 118 |
| Flk1-Cre | E10.5 | 8 (36.4%) | 4 (18.2%) | 3 (13.6%) | 7 (31.8%) | 22 |
|  | P1 | 12 (26.1%) | 13 (28.2%) | 7 (15.2%) | 14 (30.4%) | 46 |
| Mesp1-Cre | E10.5 | 6 (21.4%) | 11 (39.3%) | 4 (14.3%) | 7 (25.0%) | 28 |
|  | P1 | 18 (23.1%) | 20 (25.6%) | 16 (20.5%) | 24 (30.8%) | 78 |

**Supplemental Table 1. Genotypic analysis of embryos for generation of cell / tissue specific IP_3_R1 and IP_3_R2 knockout mice.** To generate cell / tissue specific IP_3_R1 and IP_3_R2 double knockout mice, male Cre^+^*Itpr1*^f/+^*Itpr2*^-/-^ mice were crossed with female Cre^-^*Itpr1*^f/f^*Itpr2*^-/-^ mice. The genotypes were first analyzed at postnatal day 1 (P1) to see whether the offspring were born at Mendelian ratios, and the embryos at E10.5 were then collected for morphological analysis.
