# Supplementary material for: Inositol 1,4,5-trisphosphate receptors are essential for fetal-maternal connection and embryo viability
Source: PLoS Genet. 2020 Apr 22;16(4):e1008739. doi: 10.1371/journal.pgen.1008739 (PMC7176088; doi:10.1371/journal.pgen.1008739)
Supplement: S2 Table — a Not investigated. (DOCX) [file pgen.1008739.s009.docx]

| Age of DKO embryos | Growth retardation | Reduced labyrinth layer in placenta | Thinner umbilical cord / Allantois | Dilated Ventricular chamber | Heart beat | Chest edema |
| --- | --- | --- | --- | --- | --- | --- |
| E9.5 | Partial | All | All | Partial | All | Very few |
| E10.5 | All | All | All | All | All | Most |
| E11.5 | All | -^a^ | -^a^ | -^a^ | None | -^a^ |

**Supplemental Table 2. Summary of embryonic phenotypes observed in IP_3_R1 and IP_3_R2 double knockout embryos between E9.5 and E11.5.**

^a^ Not investigated.
